# The nocturnal life of the great scallops (*Pecten maximus*, L.): First description of their natural daily valve opening cycle

**DOI:** 10.1371/journal.pone.0279690

**Published:** 2023-01-11

**Authors:** Elie Retailleau, Arthur Chauvaud, Gaetan Richard, Delphine Mathias, Laurent Chauvaud, Sarah Reynaud, Jerome Mars, Sylvain Chauvaud

**Affiliations:** 1 Société d’Observation Multi-Modale de l’Environnement, Brest, France; 2 Laboratoire des Sciences de l’Environnement Marin (LEMAR), UMR 6539 CNRS, UBO, IRD, Ifremer, LIA BeBEST, Institut Universitaire Européen de la Mer (IUEM), Plouzané, France; 3 IMT Atlantique, Plouzané, France; 4 Université Grenoble Alpes, CNRS, Grenoble-INP, GIPSA-lab, Grenoble, France; Helmholtz-Zentrum fur Ozeanforschung Kiel, GERMANY

## Abstract

Valvometry techniques used to monitor bivalve gaping activity have elucidated numerous relationships with environmental fluctuations, along with biological rhythms ranging from sub-daily to seasonal. Thus, a precise understanding of the natural activity of bivalves (*i*.*e*., not exposed to stressful environmental variations) is necessary as a baseline for detecting abnormal behaviors (deviations). This knowledge is also needed to reliably interpret observations of bivalve gaping behavior and associated biological processes (*e*.*g*., respiration, nutrition) acquired over time-limited periods. With this in mind, we investigated the natural daily gaping activity of the great scallop (*Pecten maximus*) by continuously monitoring 35 individuals in several individual tanks and *in situ* (Bay of Saint-Brieuc, Brittany, France) using fully autonomous Hall effect sensors. Our results revealed a circadian cycle (τ = 24.0h) in scallop gaping activity. Despite significant inter-individual variability in mean opening and cycle amplitude, almost all individuals (87.5%) exhibited nocturnal activity, with valves more open at night than during the day. A shift in light regime in the tanks triggered an instantaneous change in opening pattern, indicating that light levels strongly determine scallop activity. Based on the opening status of scallops, we also identified several gaping behaviors deviating from the regular daily pattern (lack of rhythmicity, high daytime opening), potentially reflecting physiological weakness. While further long-term studies are required to fully understand the natural activity of scallops, these findings pave the way for studies focused on the scallop response to external factors and introduce further research into the detection of abnormal behaviors. Coupling observations of diel valve gaping cycles with other daily variations in organismal and environmental parameters could help explain mechanisms driving the growth patterns of scallops observed in their shell striations. From a technical perspective, our field-based monitoring demonstrates the suitability of autonomous valvometry sensors for studying mobile subtidal bivalve activity in remote offshore environments.

## Introduction

Bivalves are a class of marine and freshwater mollusks characterized by a shell with two hinged parts (left and right valves) enclosing a soft body [1]. In keeping with a largely sedentary and filter-feeding lifestyle, most species move their valves to perform various vital functions such as respiration and nutrition by ventilating their paleal cavity, expelling feces, and releasing gametes into surrounding waters [2]. For most species, the closure of their valves isolates the soft body from the external environment and confers protection from predators and other threats [1]. Some species, such as pectinids, can perform complex valve movements to bury themselves in sediments, jump, or even swim [3]. Thus, by reflecting routine and discrete behaviors, bivalve gaping activity can potentially be used as a proxy of their biological processes (*e*.*g*., respiration, nutrition, reproduction, interaction between organisms) and responses to environmental variations.

The main valvometry methods for measuring valve opening include imaging [4], accelerometry [5, 6], and electromagnetic-based sensors using the Hall effect principle [7, 8] or electromagnetic induction between two electric coils (High-Frequency NonInvasive) [9, 10]. These technologies allow monitoring of the opening state of valves (with a low sampling rate for low-frequency movements) and discrete (*i*.*e*., punctual) behaviors such as jumps and closing events (with a high sampling frequency to detect fine-scale movements).

Studies using valvometry sensors have highlighted direct behavioral responses to environmental fluctuations in temperature [11], salinity [12], pH [10, 13] and oxygen conditions [14, 15]. Comeau *et al*. [11] found that the oyster *Crassostrea virginica* wakes up (opens valves) when temperatures exceed a certain threshold (0.2 to 4.0°C) and is completely closed below this threshold. A decrease in the seawater salinity leads to a decrease in the valve opening and number of movements in the blue mussels (*Mytilus edulis*), up to a threshold triggering a complete closure, protecting them from osmotic stress [12]. Bamber [12] explained that the salinity threshold value varies among populations and depends on the mussel’s natural environment and their adaptive evolutionary development. A drastic decrease in pH (6.2) triggers an increase in valve movements in the black clam (*Arctica islandica*), a subtidal species, likely because of a respiratory response to hypercapnia (buildup of carbon dioxide) [13]. This reaction seems to differ among species since Clement *et al*. [16] found that pH decreases down to 6.8 did not affect valve gaping of the eastern oysters (*Crassostrea virginica*). Lab and field studies have shown that eastern oysters respond to severe hypoxia by closing their valves [14, 15]. Behavioral responses (valve movements) also have been reported with exposure to toxic microalgae [8, 17], increased concentrations of suspended particle matter [18] and sedimentation [19], and exposure to low-frequency sounds (<1 kHz) [20, 21].

Based on the current literature, factors that are often associated with discrete events (phytoplankton bloom, storm, maritime anthropogenic activities) appear to induce an increase in discrete movements, particularly closure events. Such responses have been observed for Akoya pearl oysters (*Pinctada fucata*) exposed to a harmful dinoflagellate (*Heterocapsa circularisquama*) [8] and for Mediterranean mussels (*Mytilus galloprovincialis*) [17] and great scallops (*P*. *maximus*) [6] exposed to toxic *Alexandrium minutum*. An increased number of closure events also has been reported for *P*. *maximus* in response to high concentrations of suspended particles [18]. Recently, Charifi *et al*. [20] and Hubert *et al*. [21] reported that the Pacific oyster (*Crassostrea gigas*) and the blue mussel (*M*. *edulis*) react to low-frequency sound emissions by partially closing their valves.

Besides the direct response to natural or anthropogenic stressors, several studies monitoring gaping behaviors over time, have shown cyclical patterns at different time scales, linked to extrinsic factors [22–24]. Circadian rhythms (periodicity cycle “τ” = 24.0h) in valve gaping, with a clear opening difference between day and night, have been reported for many bivalves, including freshwater and marine species, in various environments from temperate to tropical to Arctic waters. Nocturnal activity (*i*.*e*., valves more open at night) has been observed for fresh water mussels (*Anodonta anatina* and *Unio tumidus*) [25], Mediterranean mussels (*M*. *galloprovincialis)* [26, 27], blue mussels (*M*. *edulis*) [7, 28, 29], zebra mussels (*Dreissena polymorpha*) [30], green-lipped mussels (*Perna canaliculus*) [31], and Arctic scallops (*Chlamys islandica*) [32]. Conversely, Schwartzmann *et al*. [33] showed that the giant clam (*Hippopus hippopus*) exhibits a circadian cycle with diurnal activity, closing its valves at night. These observed rhythms are mainly driven by the light cycle and result from strategies associated with predation or symbiotic relationships [26, 28, 33]. Nocturnal activity is often considered as a strategy for feeding while minimizing the likelihood of predation [26–28, 34], although the diurnal activity of the giant clam is probably associated with physiological oxidative stress triggered by symbiotic zooxanthellae [33]. Gaping rhythms with annual shifts have been reported for at least two species, the fan mussel (*Pinna nibilis*) and the oyster *C*. *gigas* [22, 23]. Both apparent circadian and circalunar cycles were first observed in the gaping activity of the fan mussel [35]. In a more recent study, Garcia-March [23] found seasonal activity patterns: from mid-July to early November, fan mussels opened their valves based on the position and illumination of the sun and moon, whereas during the rest of the time, gaping activity was directly influenced by current intensity and direction. These findings were supplemented by Hernandis Caballero *et al*. [24] showing that temperature regulates the switch between these seasonal trends. Oyster gaping activity seems driven by a complex association of solar and lunar cycles, exhibiting circadian and seasonal rhythms with nocturnal activity in autumn–winter and diurnal activity in spring–summer [22, 36]. Mat *et al*. [22] suggested links among the seasonal observed pattern in *C*. *gigas*, food availability, gametogenesis, and metabolic demand.

Thus, numerous relationships between bivalve gaping activity and environmental fluctuations have been elucidated and, as with a wide range of marine organisms, biological rhythms (circadian and others ranging from sub-daily to monthly or seasonal) appear to be common for many species [37, 38].

An accurate baseline knowledge of their natural activity (*i*.*e*., activity observed without any stressful environmental variations, generated by natural or anthropogenic factors) [25, 39] is therefore necessary to detect abnormal behaviors (deviation from routine behavior), which is of particular interest to study their behavioral response to external factors or to develop biomonitoring systems (*i*.*e*., monitoring bivalve health and detection of abnormal behaviors linked to potential perturbations of its surrounding environment [30, 40–43]). This knowledge is also needed to reliably interpret observations of bivalve gaping behavior and associated biological processes (*e*.*g*., respiration, nutrition) acquired over time periods of limited length (study duration shorter than the period of a biological cycle performed by an organism, *e*.*g*., less than 24h for an organism performing a circadian cycle).

The present study focuses on the great scallop (*P*. *maximus*), a subtidal bivalve (Pectinidae) of great economic importance with a large distribution in the Atlantic NE ocean, from temperate waters (Spain/Morocco) to sub-Arctic waters (North of Norway, Lofoten Island). This bivalve is known to be particularly alert with a developed sensitive system including in particular a remarkable visual acuity thanks to numerous eyes distributed all around the margin of the mantle fold [44–46]. Scallops are also able to realize complex behaviors such as swimming to avoid predators [3]. Although a myriad of aspects of its biology have been studied, including its punctual behaviors (*e*.*g*., swimming event, jumps), its gaping activity across periods of several days remains largely unknown. Technological advances have enabled transformation of valvometry sensors into non-invasive, autonomous, and field-deployable monitoring devices. These new tools appear to be particularly suitable for studying the activity of these swimming bivalves in experimental tanks and even in their natural habitat at 40m depth on sandy bottoms.

The aim of this study was to acquire a baseline understanding of the natural valve opening behavior of the great scallop. As a first approach and considering previous studies reporting daily rhythms in the activity of various bivalves, we focused on the gaping state (*i*.*e*., valve opening without discrete behaviors) of the scallop on a day-to-day scale. To monitor the daily activity of the scallops, we used recently developed valvometers (Valve-Trek; Technosmart Europe srl, www.technosmart.eu), which are fully autonomous and operate based on the Hall effect principle. A total of 35 scallops were monitored through tank and field experiments. This work is a necessary step for future studies focusing on the effect of environmental disturbances on the behavior of this species.

## Materials and methods

### Sensor

**Fig 1:**
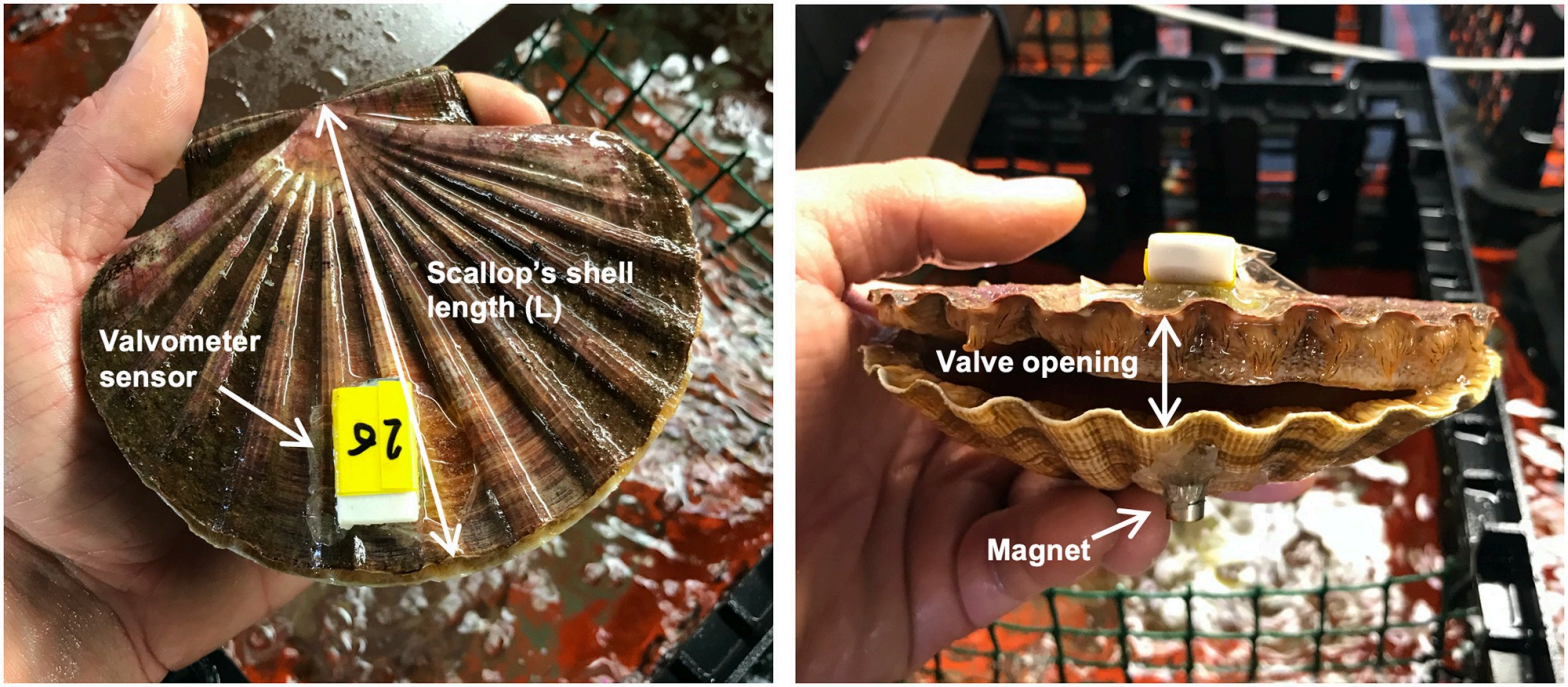
Positioning of Hall effect sensors and magnets on scallops. Schematic view of a scallop equipped with a valvometer sensor.

Autonomous valvometers (Valve-Trek; Technosmart Europe srl, www.technosmart.eu) were used to measure the valve opening (maximum distance between two valves, ventral edge) activity of the great scallops. Including Hall effect sensors, the valvometers were attached (glued with epoxy resin; epoxy express 1 min, Loctite^®^) to the left valve of the scallops to record magnetic field variations of magnets placed (glued with epoxy resin; epoxy express 1 min, Loctite^®^) on the right valve (Fig 1). The sampling frequency of the sensors was set to 2Hz to record low-frequency movements of the bivalves (variation in their opening status). This sampling rate (0.5 s) also allowed for the recording of punctual behaviors produced by faster movements of their valves (*e*.*g*., jumps, partial closures). The sensors and magnets were positioned uniformly on all individuals with the valvometer fixed at the ventral edge on the antero-posterior axis of the scallops (Fig 1). The size of these sensors (length: 22mm; width: 13mm; height: 8mm; weight: 3.5g) was consistent with potential epibionts of scallops such as the slipper-limpet (*Crepidula fornicata*).

### Tank experiment

**Fig 2:**
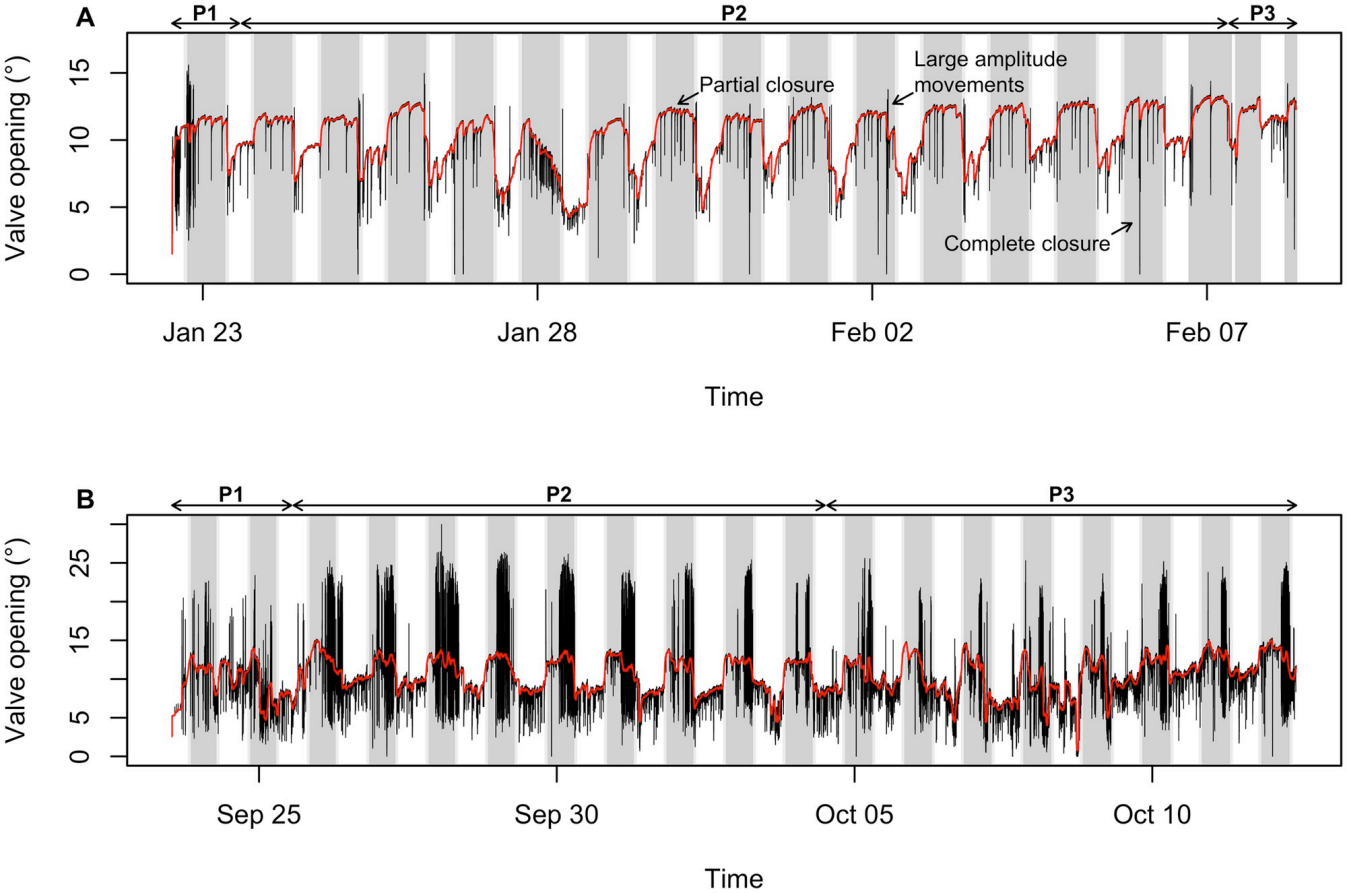
Temporal variation in gaping activity for two scallops monitored (A) at the Tinduff Hatchery and (B) in the Bay of Saint Brieuc. The black lines indicate the raw opening values (°) recorded by the valvometers, calibrated and converted into opening angle. The red lines represent the “base opening” calculated by applying a median filter of 1h duration on these data. The more shaded parts represent the night periods, and the slightly shaded parts indicate the transition phases. The different stages of the experiments are noted above the graph: P1, periods of presumed acclimatization; P2, periods used for analysis; and P3, periods with a change in light cycle for the scallop monitored in tanks and the period biased by sea conditions (cage movements) during the *in situ* monitoring. The arrows in (A) indicate different types of punctual behavior performed by the scallops.

In January 2021, ten great scallops (adult; size: 96.14mm ± 2.80, antero-posterior axis) were harvested by scuba diving at the Auberlach cove site (48.333N -4.415W; Bay of Brest, France) at depths ranging from 2 to 5m (Lowest Astronomical Tide; maximal tidal range: 6m) and transferred to Tinduff Hatchery (Plougastel-Daoulas, Brittany, France). Individuals were first placed in a supply tank for seven days of acclimatization, during which they were equipped with valvometers. The scallops were then distributed in individual tanks (one scallop per tank) for 18 days. All tanks used had a surface area of 0.78m2 (length: 130cm; width: 60cm; depth: 40cm; 312L) and a sandy bottom. Water of the tanks was supplied and renewed at 0.2L/min (complete renewal in 24 hours) *via* a continuous flow-through system with water pumped in the Bay of Brest and filtered at 1μm. Scallops were fed to satiation during the acclimatization period (supply tank) and during the first ten days of the monitoring (individual tanks) with the diet commonly used by scallop farmers: 10 billion cells per day per individual of a mixture of live diatoms (*Chaetoceros muelleri*, size: 5–8μm) and flagellates (*Tisochrysis lutea*, size: 3–5μm; *Diacronema lutheri*, size: 2–4μm) [47]. These food intakes were provided continuously by mixing the phytoplankton algae mixture with the filtered water supply in an upstream tank, at a concentration of 30 cells μL^-1^. After 10 days of the experiment, the food supply was stopped to explore whether scallop valve activity changes with food availability as might occur in their natural environment [48]. Each tank was covered with a canvas and was illuminated separately by a LED spotlight bar (NICREW Classic LED Plus 120-150cm, 1150lm, white light mode) placed 1m above the water surface. The spectrum of the emitted light covered all wavelengths in the 380-780nm range. The photoperiod was fully controlled using a light timer (Single channel LED light timer pro, NICREW) in order to simulate the natural light cycle at the time of the experiment for the area where the scallops were harvested (Bay of Brest). Thus, the light cycle was set with a period of complete darkness from 5:30pm to 8:30am, a gradual light increase from 8:30am until 9:00am, and a light decrease from 5:00pm until 5:30pm (Fig 2). This light cycle was monitored in each tank using relative light level sensors (Temperature/Light 64K Data Logger, HOBO Pendant ^®^), which were placed at the bottom of the tanks (in the center) and mounted horizontally so that the sensor pointed straight up towards the light. The light intensity recorded by these sensors during the hours of maximum illumination (9:00am to 5:00pm) varied between 35 and 40 lux. Salinity, pH, oxygen, and temperature were also monitored and controlled to guarantee a stable environment. During the experiment, the water was maintained fully oxygenated (100% O_2_ saturated) and at a constant temperature of 12°C (continuous monitoring). Salinity and pH were also constant with values of 31.2psu and 8.2 respectively (one point measurement per day using a pH 3310 probe, WTW^™^). Because of the water source irrigating the tanks (directly pumped from the bay), these latter parameters are similar to those of the seawater adjacent to the hatchery.

### Field study

In September 2021, 25 adult great scallops (size: 97.32mm ± 5.09, antero-posterior axis) equipped with valvometers were deployed for a period of 19 days within the Bay of Saint Brieuc (Brittany, France), about 16.5 nautical miles from the coast (48.895N -2.580W). These individuals were collected nine days earlier by scuba divers at the Porz ar Birnec site (48.744N, -2.941W; Bay of Saint Brieuc) at depths ranging from 15 to 20m (LAT; maximal tidal range: 12m) and then kept in tanks (Plouezec, Brittany, France) to equip them and wait for the deployment mission. During this period of captivity (8 days), scallops were isolated in 0.16m^2^ compartments within closed-circuit tanks with a constant temperature of 16°C. They were not fed. Scallops were then deployed *in situ* from a boat, in individual hard-bottom PVC cages of 0.215m^2^ (length: 50cm; width: 43cm; height: 40cm) separated by 10m, allowing each individual to be considered independent during the field experiment (no interaction possible between the sensors and between individuals). Cages were selected based on operational deployment constraints (deployment from a boat, without diver intervention). The hard bottom was not ideal for scallops, preventing them from burying themselves in the sediment. However, since the sides of the cages consisted of wide mesh nets (35mm side), it is unlikely that they affected the exposure of the scallops to local current and natural light. With a soft substrate and depths of 35–40 m, the study site is part of one of the largest source areas of scallops in France.

### Data processing

#### Valve opening

Once the scallops were retrieved after 18 and 19 days from tank and field experiments respectively, all recorded time series were calibrated to convert magnetic field intensity variations emitted by magnets (mV) into accurate valve opening distances (mm). Following a standard procedure, this calibration step was performed after the deployment by computing individual calibration curves linking recorded valvometer values to a set of known inter-valve distance (0, 5, 10, 15, 20, 25, 30, 35mm) [11]. This procedure was repeated for each individual separately. We first severed their adductor muscle. Then, the Hall voltages corresponding to the different calibration distances were measured, by inserting a series of calibrated wedges between their valves. To eliminate inter-individual differences from variations in shell size, we converted these calibrated valve opening data (mm) into gape angles (θ in degrees) using the following equation (derived from [7, 11]):

$$\theta ={cos}^{-1}\left(1-\frac{W^{2}}{{2\times L}^{2}}\right)\times \frac{180}{\pi }$$ (1)

where W is the valve opening (mm) and L (mm) is the scallop’s shell length (Fig 1).

The base opening, defined as the opening of the scallop without punctual behaviors involving rapid movements (*e*.*g*., jumps, partial closure, expulsion of feces), was calculated by applying a median filter of a duration (length of the sliding window) of 1h (7200pts) on the calibrated time series. Using a sliding window on the signal, this statistical filter smoothes data by replacing the center point of the window with the median of the signal values on the entire window. A duration of 1h allowed for effective filtering of punctual behaviors and was consistent in terms of time scale with the study objective of acquiring information on the gaping state of the great scallops through the day. Time series data were then plotted with different time scales for exploration and analysis.

#### Frequency analysis

Spectral analyses were performed with MATLAB (version 2020a, The MathWorks Inc.). To study the occurrence of cycles in the behavior of the scallops (valves opening), a frequency analysis (using Fourier transform and spectral analysis tools) was performed on each base opening time series (filtered time series). By using Fourier transform, we computed projection of a real signal consisting of infinite cosine and sine complex functions (in the frequency sampling range). Thus, we decomposed a real (or complex) signal into a series of sinusoids at different frequencies. At each frequency, the physical result of the Fourier transform can be seen as a magnitude representing the amount of that frequency present in the original function. A power spectral density, representing the frequency distribution of the signal power, was then computed for each scallop. This approach allowed us to observe the presence of cycles in a time series and to define their frequency and associated period [49]. Slow rhythms with a period greater than two days were not considered here.

#### Statistical analysis

Statistical analyses were performed with R (version 4.0.4, R Core Team). We aimed at assessing whether scallops vary their base opening (*i*.*e*., opening status) according to the phases of the day (dusk, night, dawn, day). The base valve opening was first aggregated (median) by phases of the day. For the field study, these phases were defined from the calculated solar declination values for the study area. The phases of transition (dusk and dawn) were the nautical twilight (solar declination: 0° to -12°). For the tank experiment, the night is the period of total darkness, the dusk and dawn are the periods of change of luminosity and the day is the period when the light is maximum. Since the experimental conditions and scallop populations are different between the two experiments (lab, field), statistical analyses were applied separately (data not pooled). Thus, linear mixed models (“nlme” R package) [50] were constructed (for lab and *in situ* experiments) with the base opening of the valves as explained variable, the period of the day as explanatory variable (fixed effect), and the different individuals as a random effect on the intercept to account for inter-individual variability in average opening of scallops:

$$y_{ij}=\alpha +\;a_{i}+(\beta {)\;x}_{ij}+\varepsilon_{ij}$$ (2)

with y_ij_ as the base opening of the valves for individual i over the period j, α the intercept, a_i_ the random intercept for individual i, β the coefficient of the predictive variable, x_ij_ the period of the day, and ε_ij_ the residual for the period j and individual i. Because x_ij_ is a categorical variable (*i*.*e*., dusk, night, dawn, day), the slope in practice describes differences among the categories.

A first order autoregressive correlation structure was included within models, to account for temporal autocorrelation associated to the repeated measure design (data measured on the same individual over time).

The models likelihood and the interest of their random effect were confirmed by comparing the mixed models to simple linear models (using Generalized Least Squares) and null models (*i*.*e*., *y*_*ij*_ = *α* +*ε*_*ij*_) using likelihood ratio tests and Akaike information criterion (AIC [51]; S1 Table).

Assumptions of normality were confirmed through the visual examination of the distribution of residuals and Q–Q plots. One-way ANOVAs were used to test the effect of phases on the opening angle of scallops for each experiment. Then Tukey HSD post-hoc tests were employed to determine pairwise differences between each of the phases. Statistical significances were set at 0.05.

Full statistical results can be found in the Supporting information.

## Results

### Data acquisition

#### Tank experiment

A total of 18 days was recorded for all monitored scallops (N = 10). All individuals exhibited burying behaviors during the day following deployment (period P1, Fig 2A). We noticed that a technical problem occurred at the end of the experiment (the last day), with an extended power failure that left the lights of seven tanks out (only the lights, oxygen supply and water flow were not affected) during the day phase from 10:25am to 7pm before they were switched back on and resumed the programmed rhythm (period P3, Fig 2A). The first (P1, Fig 2A) and last (P3, Fig 2A) days of the experiment were therefore not taken into account in the analyses of the natural behavior of the scallops, but the latter provided an interesting opportunity to study the influence of a sudden light switch on their gaping activity.

#### Field study

Of the 25 scallops deployed, three died during the monitoring period. Two died directly after the deployment, and the third died ten days later. This third individual showed an atypical opening activity from the beginning of the experiment; thus, its time series was compared with those of the other individuals but not considered in the analyses of rhythmicity. All other scallops exhibited unrhythmic and asynchronous activity during the first two days of monitoring, performing high-amplitude rapid movements (jumps) throughout the whole day (Fig 2B). This period (P1, Fig 2B) probably reflects their acclimatization to a new environment and was not accounted for. Rough seas led to movement of the cages 11 days after deployment until the end of the experiment (P3, Fig 2B). Thus, data for a total of nine days for 22 great scallops were usable for analyses (period P2, Fig 2B).

### Valve opening behavior

**Fig 3:**
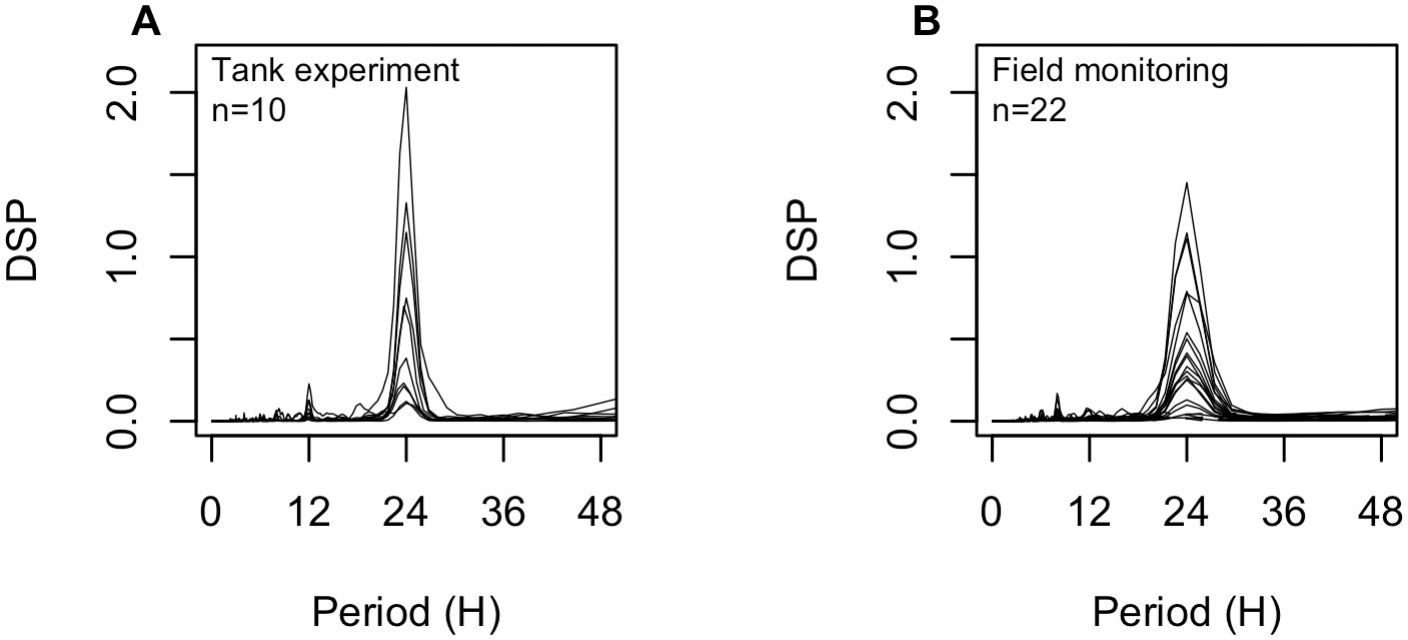
Power spectral densities computed for each scallop monitored (A) at the Tinduff Hatchery (tank experiment) and (B) in the Bay of Saint Brieuc. The lines represent the individual power spectral densities.

The spectral analyses of valve opening behavior showed that 100% (10/10) of the scallops placed in tanks and 81.8% (18/22) of those monitored *in situ* displayed a 24-h cycle (main component in the range 0–48h). Power spectral densities indicated that these cycles were more or less marked depending on the individual (variability in the importance of the 24-h frequency component among individuals; Fig 3). In the laboratory experiment, all individuals maintained the completion of this cycle after the cessation of food intake, from the 10th day to the end of the experiment (except on the last day when the light cycle shift occurred). Thus, valve opening data acquired under both feeding conditions were pooled for the following analyses.

**Fig 4:**
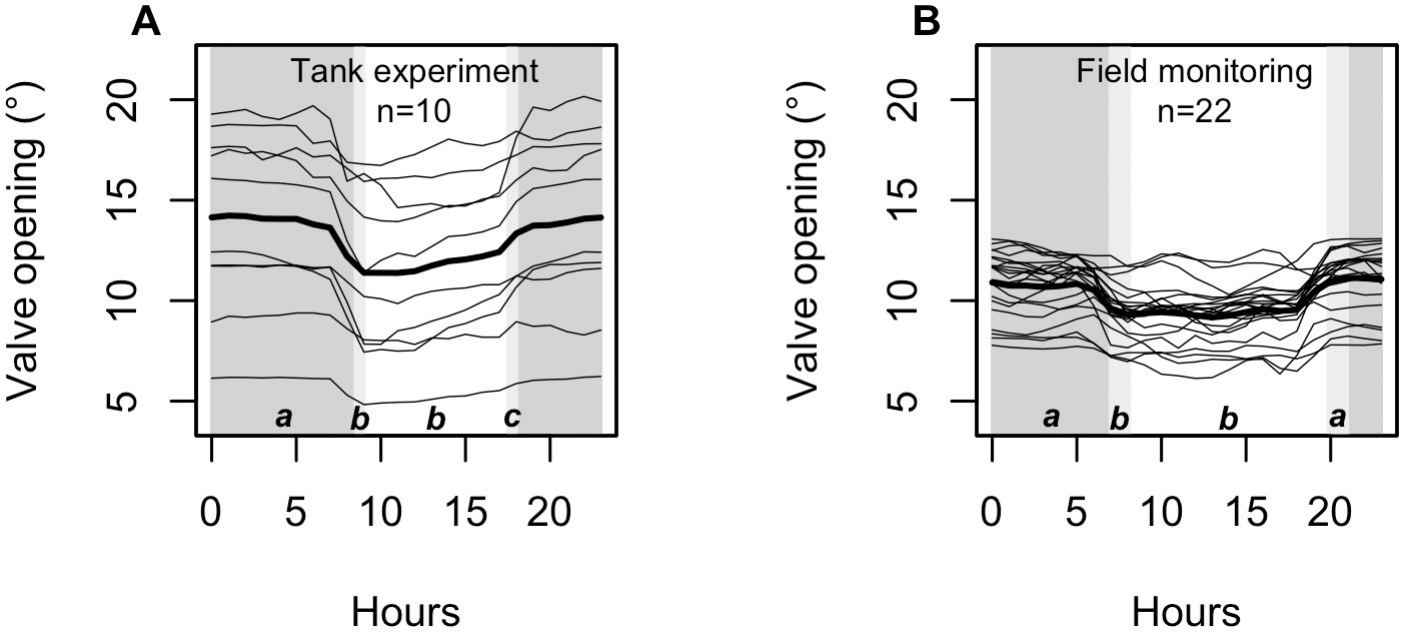
Pattern of daily mean opening of scallops monitored (A) at the Tinduff Hatchery (tank experiment) and (B) in the Bay of Saint Brieuc. Thin lines represent mean base openings per individual, and the bold line is the average of all individuals (considering all the data in the analyzed periods). Shaded parts indicate transition phases (light grey) and periods of total darkness (dark grey). The letters at the bottom of the graph indicate the result of the linear mixed model, with different letters between the groups (phases of the day) indicating significant differences.

The average opening of scallops was 12.95° (± 4.24 [5.71–18.01]) in tanks and 10.11° (± 1.35 [7.35–12.09]) for those deployed in the Bay of Saint Brieuc (Fig 4). Significant opening differences were found depending on the phases of the day (linear mixed model *p*-value < 0.001 for both experiments; Fig 4; S2 Table). Scallops monitored *in situ* were significantly more open during dusk and night than during dawn and day (Fig 4). Scallops at the Tinduff Hatchery (in tanks) were significantly more open at night. Dusk appeared as an intermediate phase with significantly larger valve openings than during dawn and day (Fig 4). Looking at the hourly average individual opening, we observed *in situ* that scallops closed their valves just before dawn and opened them just before dusk (Fig 4). Those monitored in the tanks also closed their valves just before dawn but opened them during dusk (Fig 4).

The observed 24-h opening cycle therefore seems to be correlated with the light regime. Interestingly, the seven scallops which endured the power cut (change in light regime) all responded the same way. All individual shifted their opening cycle at the same time following the abnormal light cycle, without transition phase (Fig 2A).

Although within each experiment most individuals exhibited a similar pattern of opening activity, there was significant inter-individual variability in mean opening angle and 24-h cycle amplitude (magnitude of variations in opening during the day). Indeed, the likelihood of models is significantly improved when individuals are included as a random component on the intercept to account for this inter-individual variability in mean opening angle (S1 Table). The average individual opening extended from 7.35° to 12.09° for the Bay of Saint Brieuc and from 5.71° to 18.01° for the Tinduff Hatchery. The average day/night opening deviations were 2.24° ± 1.11 [0.88–4.22] in tank and 1.40° ± 1.06 [-0.30 to 3.50] *in situ* (1.73° ± 0.85 [0.44–3.50] when the four scallops not showing a 24-h cycle were removed).

**Fig 5:**
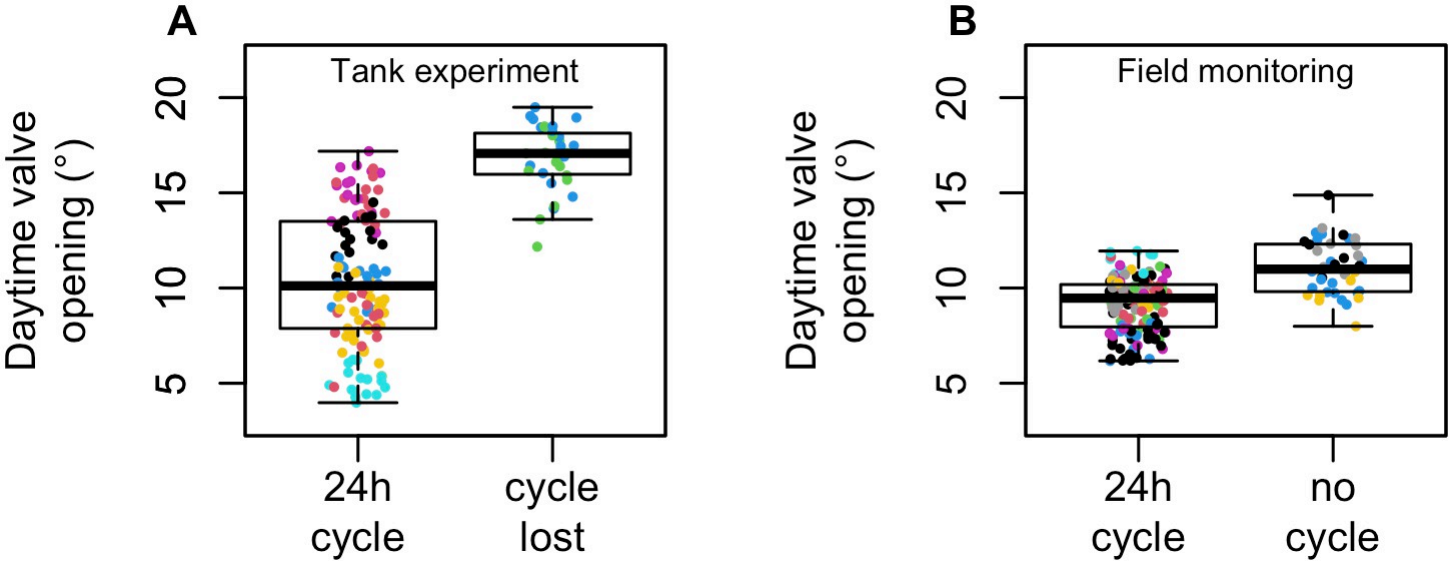
Comparison of the mean opening angle during the day time between scallops exhibiting a 24-h cycle and those without (4 scallops during the field experiment) or those having lost their cycle during the experiment (2 scallops during the tank experiment). Each point corresponds to a day of monitoring for a given scallop. The colors represent the different scallops. The black dots in the “no cycle” group of the field monitoring represent the scallop that died during the monitoring.

Despite this inter-individual variability in mean opening and the importance of the daily cycle, some scallops could be distinguished. Four scallops showed no day/night cycle during the experiment in the Bay of Saint Brieuc (inverted day/night opening deviations). Two other individuals also gradually lost their 24-h activity cycle during the last days of the Tinduff Hatchery experiment. These individuals were among those with the highest mean diurnal opening within each site (Fig 5). Similar patterns were observed for the scallop that died 10 days after deployment in the Bay of Saint Brieuc. All these individuals showed a lack of rhythmicity and high valve opening values are measured, independently of the phase of the day (Fig 5).

## Discussion

### Light-induced daily opening cycle

This study shows for the first time a circadian cycle (τ = 24.0h) in the valve opening activity of the great scallop *P*. *maximus*. Almost all individuals monitored both in tanks in winter (under controlled conditions) and *in situ* (in an offshore site, at a depth of 35-40m) in the late summer showed nocturnal activity, with valves more open at night than during the day. The synchronicity of scallop gaping behavior with the day/night cycle, combined with the change in the opening patterns following changes in light exposures in the tanks, suggest that ambient light strongly determines scallop activity. Similar to other pectinid species, the great scallops have a well-developed visual system comprising up to 200 eyes distributed all around the margin of the mantle fold [46, 52]. Containing a multilayered concave mirror (to focus light) and a double-layered retina (with photoreceptors), their eyes are suited to capture the wavelengths of light that penetrates the scallop’s habitat [46]. The importance of daylight has already been reported for this species. This factor has been correlated with physiological and behavioral changes. The increase of the photoperiod and temperature in spring induces the development of their gonads, preparing the scallops for reproduction [53, 54]. In particular, the photoperiod seems to influence the oocyte growth [55]. In addition, the light affects the vertical distribution of *Pecten maximus* larvae [56].

Although scarce, information on pectinid behaviors available in the literature is consistent with the current findings. Salanki [57] reported a circadian rhythm for *Pecten jacobaeus* that depends on illumination and can be shifted by lengthening the periods of light or darkness [25]. More recently, Tran *et al*. [32] observed a circadian rhythm with valves more open at night than during the day in the Arctic scallop, *C*. *islandica*. Although specific data are not published to date, Tran *et al*. say in their study that this behavior is similar to that of lower latitude counterpart species in Norway (Tromso, 69.63N) and Russia (Murmansk, 68.95N) and of counterpart species from temperate zones, such as the scallop *Chlamys varia* [32]. Similar behaviors have also been reported for mussel species. Circadian (nocturnal) rhythms in valve activity, with valves open more widely at night than during the daytime, have been observed for Mediterranean mussels (*M*. *galloprovincialis*) [26, 27, 58], blue mussels (*M*. *edulis*) [7, 28, 29], zebra mussels (*Dreissena polymorpha*) [30], and green-lipped mussels (*Perna canaliculus)* [31]. As in the current study, mussels have been found to respond to light intensity (synchronized shift of the circadian opening cycle with change in light cycle), and the light regime is considered as one of a main factors determining their circadian rhythm [26, 28].

The occurrence of apparent behavioral rhythms can involve two underlying mechanisms: (*i*) a functional clockwork physiological system (endogenous mechanisms) synchronized by external cues called zeitgebers [59]; or (*ii*) a phenomenon known as “masking,” an adaptive strategy in which animals respond directly to external cues [60]. Although a masking effect cannot be excluded, certain observations made during the current study seem to indicate that the apparent gaping rhythm arises from a weak endogenous clock (reduced degree of self-sustainment of an endogenous oscillator, [61]) driven by the light cycle, which represents an environmental zeitgeber. Indeed, the accidental change in the light regime enforced during the tank experiment was immediately followed by a shift in scallop activity without any transient desynchrony. This serendipitous observation reflects a wide entrainment in the synchronized state and a short resynchronization time after a zeitgeber phase shift, which are the main characteristics of the weak endogenous clock phenomenon [61]. In addition, we observed high inter-individual variability both in the tanks and *in situ*, with some individuals displaying strong rhythmic behavior and others showing very weak or even absent rhythmic behaviors. This divergence could be interpreted as another argument for the existence of a weak oscillator. The oscillator would be exceptionally weak for individuals with a low 24-h activity cycle, and their behavior would be determined mainly by exogenous factors [22]. Similar clues to the existence of a weak oscillator have been reported for the oyster *C*. *gigas*, including a quasi-simultaneous change in light and gaping activity cycles as well as strong inter-individual variability [22]. Mat *et al*. [22] suggested that this weak endogenous oscillator would certainly confer plasticity on this species, partly explaining its adaptability to various environments. Indications of such a mechanism also have been found in *C*. *islandica*, which maintains its gaping behavior during the polar night [32, 62].

### Adaptive significance of the observed behavior

Whether generated by a masking or through an endogenous phenomenon entrained by zeitgebers, the rhythms are considered adaptive, allowing individuals to adapt and synchronize themselves to their environments [22, 23, 26, 32, 63–65]. Given that marked cycles were observed for almost all individuals deployed in the Bay of Saint Brieuc, under conditions limited by various operational constraints (cages with a hard substrate), we can hypothesize that the underlying mechanism regulating the daily valve gaping rhythm must be robust. Comeau *et al*. drew a similar conclusion for *M*. *galloprovincialis*, detecting comparable rhythms that persisted in laboratory individuals subjected to batch-feeding and flume vibrational noises [58] and in a field study throughout a period of considerable change in oceanographic conditions (river discharge that suddenly decreased salinity and increased chlorophyll concentrations) [27].

With regard to adaptive significance, it is generally thought, in particular for mussel species, that nocturnal gaping is part of a strategy to feed while minimizing threat of predation by visually oriented predators [26–28, 34]. Although not quantified, large amplitude movements have been quasi-systematically observed at the end of the night and at dawn both in tanks and *in situ* (see Fig 2). This observed behavior was much more pronounced in scallops deployed *in situ*, performing many large amplitude movements during the second part of the night (Fig 2B). It is assumed that scallops dig into the sediment and adjust their position at the end of the night to hide from predators. Deployed on hard substrate, those monitored in the Bay of Saint Brieuc were potentially trying to bury themselves without success. These hypotheses on the role of the observed behaviors are consistent with the overall anti-predator strategy developed by pectinids and in particular by *P*. *maximus*. Adaptations in morphology and lifestyle in bivalves have a critical impact on their survival [66]. The great scallop shows many adaptations against predation pressure [66]. For instance, in addition to their remarkable visual acuity [44–46], they have a directional sensitivity to water-borne vibrations (statocysts on the edge of the mantle and an abdominal sense organ) [67, 68] and chemoreception ability [69, 70], allowing for predator detection. They also acquired the ability to swim actively to avoid attacks [3, 69, 71].

This hypothesis (predation avoidance) regarding the significance of the observed behavior cannot be verified by this study. The day/night gaping cycle could also be a response to other environmental factors potentially correlated to the daylight cycle such as food availability and oxygen concentration in sea water. However, the involvement of these factors seems unlikely since scallops monitored in the lab exhibited marked gaping cycles while oxygen was maintained at 100% saturation and maintained these cycles while food availability conditions were expected to change.

### Atypical behaviors

Considering the nocturnal activity of the scallops reported for almost all of the individuals monitored as “normal behavior”, some scallops in both the tank and field studies showed “atypical” opening activities, with a lack of rhythmicity. Of interest, the scallops that did not exhibit or that lost their 24-h cycles were also among those with the highest mean diurnal opening within each site. It is therefore possible that these individuals were no longer capable of closing during the day phase. Scallops have two types of muscles that allow to close their valves which are naturally opened *via* a hinge ligament: (*i*) the phasic adductor muscle, composed of cross-striated fibers, responsible for rapid valve closure; and (*ii*) the tonic adductor muscle, composed of smooth fibers, allowing for prolonged valve closure or low-energy maintenance of a constant valve opening [72]. An inability of scallops to contract their energy-efficient tonic adductor muscle would reflect severe fatigue and a critical state. Interestingly, the scallop which died ten days after its deployment in the Bay of Saint Brieuc, had the highest opening values during day phases. A similar phenomenon commonly referred to as “scallop yawning” is observed when scallops are held out of the water for too long and cannot stay closed. The day/night opening deviation and the diurnal mean opening thus could be promising indicators of significant weakness. The atypical activities described here could have been caused by stress from the different manipulations of the individuals (capture, sensor attachment, deployment). Furthermore, most of the individuals without observed circadian cycles were among those monitored *in situ*, under conditions limited by various operational constraints (cages with a hard substrate). Further valvometry studies involving a description of the physiological status of the monitored individuals would be required to confirm these assumptions. Indeed, the lack of a diel cycle observed for some individuals may also simply reflect inter-individual variations. Longer deployments would allow to further quantify the range in this inter-individual variability [73].

### Implications and perspectives

This study provides a first description of the natural gaping activity of scallops that could be qualified as a “behavioral norm”. It should be noted, however, that we only explored the gaping behavior of scallops on a day scale. It is possible that scallops respond to other factors over longer time periods such as season or year, as has been shown for other bivalves (oysters [22]; fan mussels [23]). Further long-term studies are needed for a full understanding of the natural gaping activity of scallops. This study also focuses on only two populations of scallops coming from different locations on the Brittany coast (Bay of Brest, Bay of Saint Brieuc). Given the wide distribution of the great scallop, additional studies of other populations are needed to extend these results to the species level.

These initial findings constitute essential knowledge for future studies focusing on the effect of external factors on scallop behavior and biomonitoring. According to some authors, natural daily variations in valve opening can mask the reaction of bivalves to environmental disturbances [26]. Furthermore, knowledge of the natural activity of scallops not exposed to stressful environmental variations, allows the detection of behaviors that deviate from this framework. Atypical behaviors, potentially reflecting physiological weakness, were thus detected in this study through indicators based on the opening status of the scallops (rhythmicity, daytime opening). These initial observations open the way to further research on the detection of abnormal behaviors. From this perspective, it would be interesting to look at the occurrence of discrete behaviors, such as jumps, swimming events, and partial or complete closures over time. In addition to methodological challenges related to behavior detection, many fundamental issues need to be addressed before considering biomonitoring of scallops, such as the quantification of inter-individual variability, the influence of external factors on gaping activity (*e*.*g*., food availability, current, concentration of suspended particles), or the phenomenon of habituation to stress as observed in mussels exposed to repeated sound exposures [21].

Coupling observations of diel valve gaping patterns with other daily variations in organismal (such as clearance and respiration rate or energy expenditure) and environmental parameters could help explain mechanisms driving the daily growth observed in shell striations (daily *striae*) and associated seasonal variations [48, 74]. The formation of these daily shell increments may be influenced by several external and internal factors such as temperature, photoperiod, food availability and physiological status of the animal (annual oscillation of reserve storage and energy allocation for reproduction and somatic growth [54, 75]).

The results of this study may also be of interest to the farming industry (hatcheries, ponds). The importance of photoperiod in the development of scallop gonads has already been demonstrated and is commonly incorporated into standard conditioning practices [47, 53, 76]. This study reinforces recommendations made on this factor, highlighting the need for precise light control, since the photoperiod directly affect scallop gaping behavior that could potentially influence their metabolism and growth.

Finally, to our knowledge, this study is the first *in situ* monitoring of bivalve behavior *via* the use of autonomous valvometry sensors in an offshore site (about 16.5 nautical miles from the coast). Despite operational constraints, this study demonstrates the feasibility of using this type of sensor to study the activity of subtidal bivalves in remote environments, thus offering promising technical perspectives to investigate, for instance, the effect of anthropogenic offshore activities on bivalves under real conditions.

## Supporting information

S1 Table Test of the likelihood of the mixed models used and of the interest of their random effect component. Comparison of the mixed models with simple linear models (using Generalized Least Squares) and null models using likelihood ratio tests and information criterion (AIC, BIC).
(DOCX)

S2 Table Results of one-way ANOVAs applied to test the effect of phases of the day on the opening angle of scallops monitored in tanks and *in situ*. (DOCX)
