# Supplementary material for: The nocturnal life of the great scallops (Pecten maximus, L.): First description of their natural daily valve opening cycle
Source: PLoS One. 2023 Jan 11;18(1):e0279690. doi: 10.1371/journal.pone.0279690 (PMC9833516; doi:10.1371/journal.pone.0279690)
Supplement: S1 Table — Comparison of the mixed models with simple linear models (using Generalized Least Squares) and null models using likelihood ratio tests and information criterion (AIC, BIC). (DOCX) [file pone.0279690.s001.docx]

| **Lab experiment** | | | | | | |
| --- | --- | --- | --- | --- | --- | --- |
| Model: | | Degrees of freedom | AIC | BIC | Test | *P*-value |
| **1** - Null model | $y_{ij}= \alpha+\varepsilon_{j}$ | 2 | 3264.137 | 3272.828 |  |  |
| **2** - Linear Model using Generalized Least Squares | $y_{j}= \alpha+(\beta{) x}_{j}+\varepsilon_{j}$ | 5 | 2139.863 | 2165.937 | 1 vs 2 | < 0.001 *** |
| **3** - Linear Mixed-Effects Models  (intercept) | $y_{ij}= \alpha+ a_{i}+(\beta{) x}_{ij}+\varepsilon_{ij}$ | 7 | 1986.102 | 2016.522 | 2 vs 3 | < 0.001 *** |

| **Field experiment** | | | | | | |
| --- | --- | --- | --- | --- | --- | --- |
| Model: | | Degrees of freedom | AIC | BIC | Test | *P-*value |
| **1** - Null model | $y_{ij}= \alpha+\varepsilon_{j}$ | 2 | 3144.039 | 3153.302 |  |  |
| **2** - Linear Model using Generalized Least Squares | $y_{j}= \alpha+(\beta{) x}_{j}+\varepsilon_{j}$ | 6 | 2295.734 | 2323.526 | 1 vs 2 | < 0.001 *** |
| **3** - Linear Mixed-Effects Models  (intercept) | $y_{ij}= \alpha+ a_{i}+(\beta{) x}_{ij}+\varepsilon_{ij}$ | 7 | 2167.212 | 2199.636 | 2 vs 3 | < 0.001 *** |
