# Supplementary material for: The nocturnal life of the great scallops (Pecten maximus, L.): First description of their natural daily valve opening cycle
Source: PLoS One. 2023 Jan 11;18(1):e0279690. doi: 10.1371/journal.pone.0279690 (PMC9833516; doi:10.1371/journal.pone.0279690)
Supplement: S2 Table — (DOCX) [file pone.0279690.s002.docx]

| **Experiment** | **Degrees of freedom** | **F value** | ***P-*value (>F)** |
| --- | --- | --- | --- |
| Lab experiment | 3 | 102.55 | < 0.001 *** |
| Field experiment | 3 | 62.82 | < 0.001 *** |
